# Book Reviews

**Published:** 1915-01

**Authors:** 


### BOOK REVIEWS

Books mentioned may be inspected at and ordered through this office. So far as possible, books received in any month will be reviewed in the issue of the second month following.

---

**Abdominal Operations.** By Sir Berkeley Moynihan, M. S. (London) F. R. C. S., Leeds, England. Third edition, entirely reset and enlarged. Two octavo volumes totaling 980 pages, with 371 illustrations, 5 in colors. Philadelphia and London: W. B. Saunders Company, 1914. Cloth, $10.00 net; Half Morocco, $13.00 net.

Vol. I includes General Considerations (though peritonitis, subphrenic abscess and operations of visceral ptosis are here placed), Operations upon the Stomach and the Small Intestine. Vol. II includes the Large Intestine and Appendix, Liver, Pancreas and Spleen. The general plan of the work is to describe the technic of the various operations and then to discuss sequelae, complications, mortality, relative merits of alternate operations, etc., with statistics, citations from cases of special interest and such generalizations as are based on actual experience both of the author and of other authorities. The utmost candor is manifest in these discussions, both in weighing conflicting surgical opinions and in treating the more general problems of lesions in such a way as to enlist the interest not only of the surgeon but of the internist. Indeed, we cannot emphasize too strongly the fact that this book should be studied and used as a guide by the internist who usually bears the responsibility of making the diagnosis and of deciding as to the propriety of surgical intervention, who may properly even have a voice in determining the general nature of the appropriate operation, and who certainly should observe and have a hand in the management of sequelae of a visceral nature.

We cull a few points of interest as illustrative of the value of the book both to the surgeon and to the internist. The bacterial content of the alimentary canal (3 hours after bread and meat meal to dogs) is 50,000 per m. g. for the stomach, 30,000 for the beginning of the duodenum, rises to over 100,000 for the end of the ileum, drops to 30,000 for the large intestine. After a prolonged fast, the alimentary canal is practically sterile as far as the caecum, which retains a high bacterial count, 100,000, but the lower bowel contains only 10,000 germs per m. g. Oral asepsis and sterilization of food with gastric lavage, greatly reduces the septic dangers from operations. beta napthol, salol, iodoform and actol are useless as intestinal antiseptics but isoform, given in half gram doses up to three grams in 2 to 24 hours, has produced a great reduction in bacterial colonies within 30 hours. As it is no longer procurable (on account of war?) the author employs bismuth in large doses before operation, whenever possible.

Gastro-enterostomy now has a mortality of less than 2%. Jejunal ulcer has been observed in only one case of malignant disease but in about 100 cases after gastro-enterostomy for other conditions. In 189 anterior operations, Van Roojen collected 6 cases of jejunal ulcer and in 444 posterior operations, 6 cases.

It is difficult to combine the statistics of different authorities with regard to the operative treatment of gastric cancer but we gather that the operative mortality of exploratory incision is about 10%, of gastro-enterostomy about 25% and that radical attempts while showing a greater mortality in the aggregate, have, in the later cases, been reduced to about the same rate as gastro-enterostomy. Kroenlein's and Mikulicz's statistics do not show any material prolongation of life by gastro-enterostomy; in fact the average duration of life for cases subjected merely to exploratory incision is stated to be the same as for non-operative cases—perhaps because the immediate hastening of death in about 10% counterbalances the tendency to exclude hopeless cases from any operation. On the other hand, gastrectomy affords a survival of two years as contrasted with one year for non-operative cases. We are glad to have such support of our contention that a case of gastric cancer, if operated on at all, should be subjected to radical operation.

A table—the most extensive of any of the kind that we have seen—includes 54 cases of intestinal resection, ranging from 192 to 540 c. m. Only one died shortly after operation, and only one after 3½ months. It seems almost unpardonable that in only a few instances were any investigations made of metabolism. Waste of fat and of nitrogen was shown but the ultimate results were surprisingly good, in spite of neglect of dietetic care in most cases.

Forty-four cases of rupture of intestine without external wound are cited—none due to inflation through the rectum, as in several American cases, two occurring in Buffalo.

Of 362 cases of typhoid perforation, subjected to operation, 268 died—74% (Harte and Ashhurst).

About 50 cases of pancreatic calculus have been recorded since Graaf's first report in 1667. As many as 300 stones have been found. The largest was 2½ x ½ inch (Schupmann). The author's case, 1902, was the first in which successful operation followed a pre-operative diagnosis.

---

**A Manual of Physiology, With Practical Exercises.** By G. N. Stewart, M. A. D. S. C., M. D., etc., Professor of Experimental Medicine in Western Reserve University, Clinical Pathologist to Lakeside Hospital, Cleveland. Seventh edition, with colored plate, and 467 other illustrations. Wm. Wood & Co., N. Y. 8 vo. 1156 pages. Cloth, $4.00 net.

This is a systematic treatise, ranking well above the standards of a brief text book for students and approaching those intended to record in detail, physiologic researches along specialized lines. Practical exercises, mainly vivisectional, are appended to each section. Due attention is paid to physiologic chemistry and frequent allusion is made to practical deductions, so that the book is by no means to be passed by because of the misconception that one has finished physiology and gone into practice. Sufficient emphasis has been laid on the necessity of constantly studying pathology; it is equally important for the practitioner to keep himself informed as to the normal standards of physiologic activities. It gives us special pleasure to note that, in the discussion of speech, the author has not reiterated the frequent misconceptions, especially that of confusing voice and speech, which have blemished many works on physiology. With his clear grasp of the general principles of this interesting and important function, we trust that the author will extend this section in future editions, so as to cover various languages as completely as possible.

---

**Stedman's Medical Dictionary.** By Thomas L. Stedman, A. M., M. D., Editor of the Medical Record, etc. Third edition, revised and enlarged. Royal 8vo 1079 pages, illustrated. Flexible red morocco, indexed $5.00 net. Plain $4.50 net. Wm. Wood & Co., Publishers, N. Y.

While the former edition of this book has by no means outlived its usefulness, it has been appreciated by the profession to the extent of exhausting the supply. It was, therefore, thought best, instead of reprinting the third edition from the plates of the second, and adding an appendix of new terms, to make a thorough revision and to include all new medical words that had established themselves in our vocabulary. By economizing space, this has been accomplished by making the third edition only thirty pages larger than the second. Stedman not only possesses the compilatory faculty so necessary in a lexicographer but the knack of formulating definitions that define instead of merely conforming to the negative test that they cannot be denied and he has the equally important skill of expressing meanings briefly. In spite of—or perhaps really on account of—broad and deep scholarship in languages, he has not fallen into the error of compiling terms as they ought to be; he has made a dictionary of the medical vocabulary as it is. By the inclusion of proper names and of classifications under general headings and thanks to the author's practical familiarity with medicine in general, the work is a good deal more than a dictionary of words. While making no pretense toward cyclopaedic completeness, it is an alphabetic compilation of medical facts.

---

**Rose & Carless' Manual of Surgery.** For Students and Practitioners. Ninth edition, revised by Albert Carless, M. S., F. R. C. S. 8vo. 1420 pages, with 609 cuts of the text, and 16 full page colored plates. Muslin $6.00 net. Half morocco $7.00 net. Wm. Wood & Co., Publishers, New York.

While, in a branch of medicine so thoroughly standardized as surgery, all systematic treatises must conform to the same general plan and contain, for the most part, the same general principles and opinions, this work demands the attention of the American surgeon on account of certain differences from the average complete surgery of an American author. In the first place, while of about the same size, the print is somewhat finer than in most American text books, though perfectly clear and legible. Again, much less space is occupied by illustrations. The authors have not considered it necessary to introduce photographic representations of method of tying surgeon's apron, electric light socket and cord for attachment of exploratory lamp, roller bandages used in applying splint, etc. As will be noted above, the book is far from being unillustrated. The colored plates are especially beautiful and, in general, the cuts represent typic or rare manifestations that need to be pictured. In short, so far as illustrations are concerned, quality and utility have been considered rather than quantity. And the economy of space thus secured renders the book one of the most complete and meaty treatises that have come to our hands.

The first chapter, on surgical bacteriology, infection and immunity, is by Dr. W. D'Este Emery. Following this; inflammation, use of heat, light, electricity and radium, examination of the blood (also by Emery), non-specific pyogenic infections, ulceration, gangrene, specific infective diseases, tumors and cysts, wounds. General operative technic marking approximately the end of the first fifth of the book may be considered to begin the operative portion of the book but the authors have not attempted to demarcate between surgical principles and surgical technic, so sharply as has been done by others. On the contrary, each subject has been discussed in logical sequence and with such attention to scientific principles and operative treatment as its nature demands. This seems to us the proper spirit of the broad-minded surgeon. Some books have unduly emphasized the division of surgery into theory and practice and, by so doing have implied that practice consisted in operating and that the discussion of surgical principles was a preliminary doing of chores before the real work started.

---

**Kirkes' Handbook of Physiology.** Eighth American Revision. Revised by Dr. Chas. W. Greene. 8 vo. 790 pages, illustrated with 2 colored plates and over 500 engravings in black and numerous colors. Wm. Wood & Co., New York. Extra muslin, $3.00 net.

This physiology, long a favorite with the profession, has been thoroughly revised by Greene. Of special value are the chapter on organic chemistry as applied to physiology and the many references to chemistry in the chapters on the various excretions and secretions. For example, we note the disappearance of oxygen from the gases of the alimentary canal in passing from the stomach to the intestine, the marked increase of carbon dioxide and the appearance of hydrogen and carbureted hydrogen, with a corresponding decrease in nitrogen. Throughout the work, information of this sort is given in convenient tables. The book is well balanced, thorough, and contains much statistic information which would not be missed by the casual reader but which is of the utmost value for reference.

---

**A Text-Book of Pathology, with an introductory section on Post-Mortem Examinations and the methods of preserving and examining diseased tissues,** by Francis Delafield, M. D. and T. Mitchell Prudden, M. D. Revised with the co-operation of Francis Carter Wood, M. D. Tenth edition. Completely revised. 8vo. 1,144 pages, illustrated by 14 full-page plates in black and colors, and by 695 line and half-tone cuts in black and numerous colors. Wm. Wood & Co., New York. Extra muslin, $6.00 net; leather, $7.00 net.

We feel almost a personal interest in this work, having used the first edition many years ago and having had the pleasure of reviewing one or two of the intermediate editions. The reputation of the authors and of their book, the mere fact that the tenth edition has been reached, renders it unnecessary to go into details. No attempt has been made to lend the appearance of novelty by departing from established precedents of arrangement but pains have been taken to bring the book up to the latest achievements of research. The enlistment of the services of Dr. Wood is commendable.

---

**The Heart in Early Life.** G. A. Sutherland, M. D., F. R. C. S., London, published by the Oxford University Press, American Branch, N. Y., 211 pages, illustrated, $2.00.

This is a characteristic English monograph, in which the blending of clinical experience and systematic didactic discussion, of the theoretic and the practical, is accomplished to a degree rarely attained by American authors, who tend to separate these points of view in authorship. The work is founded largely on polygraph studies but the conclusions reached are of value even to one not equipped for such investigations. The modifications of pathology symptomatology, prognosis and treatment due to differences of age are not realized so generally as they should be and the perusal of this book affords a special preparedness for the successful management of cardiac cases in children.

---

**Urgent Surgery.** By Felix Lejars, translated from the Seventh French edition by Wm. S. Dickie, F. R. C. S. Third English Impression. Vol. 1, Introductory, Head, Neck, Chest, Spine, Abdomen. Large 8vo, with 485 engravings in black and colors, and 10 full page plates. Muslin $7.00 net. Half morocco $8.00 net. Wm. Wood & Co., Publishers, New York.

Consider the following cases: 1. An expert, thoroughly competent surgeon, of wide experience in all ordinary surgical procedures, confronted with a condition so rare that it occurs once or twice in the practice of a life time, so rare indeed that the possibility of its existence probably has not occurred in making the diagnosis; 2. An equally expert and competent surgeon who realizes that he sometimes forgets details and who is conscientious to the extent of wishing to begin every operation as if he had just operated on a similar case and had thoroughly refreshed his memory of the subject; 3 A ship surgeon, two or three days or more from land, familiar with routine duties but necessarily with little opportunity for acquiring surgical technic; 4 A practitioner in a remote country district, or in a camp; 5 A young man at the beginning of a surgical career, inevitably having to learn from experience but wishing to minimize to the patient, both for the patient's sake and that of his own reputation, whatever danger might arise from lack of previous experience. Imagine any one of these suddenly confronted with a grave surgical crisis, requiring not only attention to every detail of technic, but familiarity with the principles of surgery as applied to a particular case, the prompt direction of assistants, preparation of the patient, operating room and instruments. We ought not even to use the word imagine without qualification for none of these cases are imaginary or even infrequent in the aggregate.

Now, conceive a logically arranged, carefully indexed work, perfected through seven editions, copiously illustrated, with every detail worked out for the operator caught in an emergency and, indeed, with schedules of necessaries and general advice to establish a state of preparedness for emergencies, and you have the nearest approach possible to a solution of the above problems. Such a book is a factor in diminishing mortality.

---

**A Manual of Diseases of the Nose, Throat, and Ear.** By E. B. Gleason, M. D., Professor of Otology in the Medico-Chirurgical College, Philadelphia. Third edition, thoroughly revised. 12mo. of 590 pages, 223 illustrations. Philadelphia and London: W. B. Saunders Company, 1914. Cloth, $2.50 net.

This book, while of moderate size, is systematic, thorough and well written in condensed style. The present edition has been revised to date. A valuable part of the work is an appendix in 33 pages of small type, giving formulae applications, sprays, etc., as well as appropriate general internal prescriptions, in the apothecary's system.

---

**Local and Regional Anaesthesia, including Analgesia.** By Carroll W. Allen, M. D., of Tulane University, New Orleans, with an introduction by Rudolph Matas, M. D., of Tulane University, New Orleans. Octavo of 625 pages with 255 illustrations. Philadelphia and London: W. B. Saunders Company, 1914. Cloth, $6.00 net; half morocco; $7.50 net.

That a technical subdivision of a branch of medical practice could be extended to occupy a large volume is one of the most interesting indications of the development of the healing art. Spinal, epidural, "paravertebral," "parasacral," dental anaesthesia and that for the eye, ear, nose and throat, are given special consideration. The regional anatomy of various nerves is depicted so clearly as to prevent delays and accidents due to faulty conceptions of anatomic relations. In fact, so far as we can judge, every problem either involving general scientific principles, or technic, or after effects is duly considered and any by-path of inquiry is followed to the end.

---

**Morris's Human Anatomy.** Edited by C. M. Jackson, M. S., M. D., Minneapolis. P. Blakiston's Son & Co., Philadelphia. 1539 pages, 1182 illustrations, 358 in colors, one volume cloth, $5.00. Also published in sections as follows: Morphogenesis, Osteology, Articulations $1.50; Muscles, Blood Vascular System, Lymphatic System $2.50; Nervous System, Special Sense Organs $2.00; Digestive System, Respiratory System (sic), Skin, Mammary and Ductless Glands Urogenital System (sic) $1.50; Clinical and Topographic Anatomy $1.50; each fasciluclus with index.

This work is written by twelve associates, English and American, including Dr. A. T. Kerr of Ithaca. The B. N. A. system of nomenclature is followed, except that corresponding anglicized terms are employed for most of the muscles and a few instances in which the B. N. A. nomenclature has been considered undesirable. Whenever necessary, the Latin term is given in brackets after the anglicized B. N. A. term. The advantage of the B. N. A. nomenclature, aside from logical reasons, consists in the condensation of the anatomic vocabulary from about 30 to five thousand words. The illustrations are clear and colors are employed to facilitate reference. The type is small but clear, and distinctions have been made to separate matter mainly required by those specializing in anatomic minutiae. The thumb index system is used to render reference to sections more convenient. In the single-volume edition, the index is complete for the whole work. Tabular arrangements are introduced wherever feasible. While this book is an unabridged anatomy and no part of the subject has been slighted, the various collaborators seem to have been actuated by the humane spirit of rendering this difficult and tedious subject as simple as is consistent with thoroughness, of reducing mental and ocular strain to a minimum, and of reflecting upon it whatever interest is possible, by allusions to its future adaptation to surgery and medicine, without going to the extent of invading other branches of medical teaching.

---

**Underworked Opportunities in Therapeutics.** By Dr. Henry R. Harrower, 880 W. 180th Street, New York.

Our attention has been called to an interesting pamphlet with the above title which has just been put out by Dr. Henry R. Harrower.

It considers the merits of organotherapy as a means of treatment that has great possibilities, many of which are overlooked in general practice. We believe that many of our readers will be glad to read this brochure, and will profit by reading it.

A copy may be had by addressing the author at 880 W. 180th Street, New York.

---

**Physicians' Daily Memorandum, 1915.** Published by the M. J. Breitenbach Co. of New York.

This is an invaluable time saver. We advise every physician to ask for a copy if he has not already received one, to make a memorandum in it of interest due or receivable, days of payment of taxes, expiration of insurance and similar business obligations, at the beginning of the year. Ample space is afforded for inserting reminders of all sorts of engagements, social, professional, of meetings of societies, etc.

---

**Physicians' Visiting List, for 1915.** P. Blakiston's Son & Co., Philadelphia, $1.25.

This is the familiar, black leather, gilt edged book arranged by Lindsay and Blakiston and now in its 64th year. It has a pocket and pencil loop and contains the usual tables of dosage, poisons, etc. It has long been a favorite with the medical profession.

---

**Progressive Medicine,** Vol. 4, December 1914. Lea & Febiger, Philadelphia and New York. Quarterly, approximately 410 pages per issue, $6.00 per annum.

The present number deals with Diseases of the Digestive Tract and Allied Organs, Kidneys, Genito-Urinary Diseases, Surgery of the Extremities, Shock, Anaesthesia, Infections, Fractures and Dislocations, Tumors, and concludes with a Practical Therapeutic Referendum. This is a standard review of medical literature, well handled by a competent staff. The editor may be pardoned for a personal allusion. In regard to Cantlie's description of tuning fork and stethoscopic location of organs, extracted from the British Medical Journal, he would refer, simply as a matter of historic interest to his own article on the general subject of Auscultatory Percussion and Allied Methods of Physical Diagnosis in the Transactions of the Medical Society of the State of New York for 1895 and published also in the defunct Medical and Surgical Reporter of Philadelphia, in the same year, with description and illustration of the tuning fork method.

